# Effect of Olive Fruit Paste on the Physicochemical Characteristics, Antioxidant Potential, Shelf Stability, and Sensorial Quality of Cheddar‐Type Cheese

**DOI:** 10.1155/ijfo/1114786

**Published:** 2026-09-02

**Authors:** Munahil Mahmood, Tahir Mahmood Qureshi, Mehrooz Ishfaq, Rana Muhammad Bilal, Muhammad Nadeem, Haile Welearegay, Mestawet Taye

**Affiliations:** ^1^ Department of Food Sciences, Cholistan University of Veterinary and Animal Sciences, Bahawalpur, Pakistan, cuvas.edu.pk; ^2^ Department of Animal Nutrition, Cholistan University of Veterinary and Animal Sciences, Bahawalpur, Pakistan, cuvas.edu.pk; ^3^ Institute of Food Science and Nutrition, University of Sargodha, Sargodha, Pakistan, uos.edu.pk; ^4^ Horticultural Sciences Department, North Florida Research and Education Center, Institute of Food and Agricultural Sciences, University of Florida, Quincy, Florida, USA, ufl.edu; ^5^ School of Animal and Range Sciences, Hawassa University, Hawassa, Ethiopia, hu.edu.et; ^6^ Food Ingredients Research Group, University College, Cork, Ireland, ucc.ie

## Abstract

The present study investigated the effect of varying concentrations of olive fruit paste on the physicochemical characteristics, antioxidant potential, shelf stability, and sensory evaluation of Cheddar cheese during storage (0–2 months, 4°C). Olive fruit paste was incorporated at different percentages (0%, 1.5%, 3%, 4.5%, and 6%), whereas date fruit paste (6%) was also added in all the treatments except T_0−_ (without olive fruit paste and date fruit paste). The pH, ash content (%), titratable acidity (%), crude fiber (%), fat content (%), and moisture content (%) of Cheddar cheese showed significant (*p* < 0.05) variations among the treatments during storage. Freshly prepared Cheddar cheese supplemented with 6% olive fruit paste (T_4_) showed the highest phenolic contents (442.47 mg/100 g GAE), total flavonoid contents (189.13 mg/100 g QE), DPPH activity (792.73 mg/100 g AAE), and FRAP activity (405.27 mg/100 g AAE). The antioxidant potential of all prepared Cheddar cheeses increased up to the 1st month of storage and then decreased after 2 months. Cheddar cheese supplemented with olive fruit paste showed a decreasing trend of total phenolics, total flavonoids, FRAP activity, and DPPH activity, whereas control cheese samples (without olive fruit paste, T_0−_ and T_0+_) showed an increasing trend of antioxidant potential throughout the storage (0–2 months). The maximum total plate counts (2.2 × 10^4^ CFU/g) and Y&M counts (1.2 × 10^1^ CFU/g) were found in T_0+_ (control cheese), whereas the minimum TPC (1.72 × 10^4^ CFU/g) and Y&M counts (6.6 × 10^1^ CFU/g) were also observed in T_0−_ after 2 months of storage. Furthermore, the sensorial properties of Cheddar cheese regarding appearance, texture, and overall acceptability decreased with the addition of olive fruit paste, whereas the flavor of Cheddar cheese was enhanced after supplementation with olive fruit paste. Based on the findings, it was concluded that freshly prepared Cheddar cheese supplemented with olive fruit paste would be nutritious and healthy due to greater antioxidant activity compared with the control cheese samples (without olive fruit paste, T_0−_ and T_0+_). Antioxidant potential, shelf stability, and sensorial quality of Cheddar cheese supplemented with olive fruit paste decreased after 2 months of storage period.

## 1. Introduction

Cheese is a good source of high‐quality protein, milk fat, minerals (phosphorus, magnesium, and calcium), and fat‐soluble vitamins that are beneficial to human health [1–3]. Cheddar cheese is one of the most popular cheese which is being consumed and produced all over the world [4]. It is a salt‐dried and hard cheese with a moisture content of 38% [5]. The ingredients used in Cheddar cheese manufacturing include lactic acid bacteria (LAB), proteins (enzymes), pasteurized milk, and salt [6, 7]. The major cultures used in the production of Cheddar cheese are *Lactococcus lactis* subsp. *lactis* and *L. lactis* subsp. *cremoris* [8, 9]. The rheological and textural properties of Cheddar cheese are influenced by several factors, including calcium and phosphorus contents, the salt‐to‐moisture (S/M) ratio, and the remaining lactose concentration [10–13].

Olive fruits are round or oval drupes and are classified into three major kinds on the basis of their weight, such as small (lower than 3 g), medium (3–5 g), or large (greater than 5 g). Most olive fruits weigh between 3 and 10 g [14, 15]. Fresh olives are not consumed (unpalatable) due to the presence of a bitter compound (oleuropein) and require processing before consumption [16, 17]. The bitterness of olives decreased as the fruit ripened, and the color of the peel progressively changed from green to light yellow, then to purple‐red, and eventually to purple‐black [18, 19]. The primary constituents of olives include water (60%–75%), reducing sugars (2%–5%), phenolic compounds (1%–3%), lipids (10%–25%), carotenoids, tocopherols, and minerals [20].

The predominant class of phenolic compounds present in olives is secoiridoids (verbascoside, ligstroside, oleuropein, and dimethyl oleuropein), phenolic alcohols (tyrosol, hydroxytyrosol, and homovanillyl alcohol), phenolic acids (caffeic acid, syringic acid, ferulic acid, *p*‐coumaric acid, hydroxybenzoic acid, *o*‐coumaric acid, sinapic acid, 3,4‐dihydroxybenzoic acid, vanillic acid, and gallic acid), and flavonoids (luteolin, cyanidin‐3‐rutinoside, luteolin‐7‐glucoside, apigenin‐7‐glucoside, cyanidin‐3‐glucoside, quercetin‐3‐rhamnoside, and rutin) [21–25]. These phenolic compounds demonstrated various biological activities, including antiplatelet, antimicrobial, wound healing, anti‐inflammatory, cardiotonic, digestive health, antiosteoporosis, antidiabetic, antihypertensive, laxative, prevention of neurological disorders, anticarcinogenic, antioxidant, and antidyslipidemic [26–29].

The date palm, or *Phoenix dactylifera* L., is one of the oldest and most valuable fruit crops due to its nutritional, religious, medicinal, and socioeconomic significance [30, 31]. Date palms are mostly cultivated in tropical regions, such as Southwest Asia, North Africa, and South Asian nations (India and Pakistan) [32, 33]. Date fruits are an important source of vitamins, easily metabolizable natural sugars, fiber, minerals, and other valuable antioxidant compounds, including phenolics and carotenoids [34, 35]. The antioxidant potential of date fruit is recognized because of the presence of various phenolic compounds, namely, procyanidins, phenolic acids (ferulic, gallic, *p*‐coumaric, and sinapic), and flavonoids (quercetin, apigenin, and luteolin) [36–38]. These compounds showed various biological activities, including nephroprotective, antioxidant, hepatoprotective, antimicrobial, anti‐inflammatory, sex hormone modulator, antidiabetic, and antitumor [39–42].

Chedid et al. [43] studied the quality and safety of some fermented dairy products in Lebanon. According to our state of knowledge, many studies used the by‐products (pomace and olive oil) of olive fruit in dairy products due to the presence of health‐promoting effects [44–46], but there is limited or lack of research regarding the utilization of olive fruit paste in dairy products. As olive fruits are not consumed fresh due to the presence of bitter compounds (oleuropein), it would be nice to add olive fruits to any dish to increase its palatability. In this regard, we added olive fruit paste into the cheese matrix, but in order to mask the bitterness of olive fruits, date fruit paste was also added into the cheese matrix at fixed quantities. Hence, the current study emphasized the impact of varying concentrations of olive fruit paste on the physicochemical characteristics, sensorial properties, antioxidant potential, and shelf stability of Cheddar cheese throughout the storage period (0–2 months).

## 2. Materials and Methods

### 2.1. Procurement of Material

Milk was sourced from Chaudhary Dairy Farm, Pirmahal (city in Pakistan). Date fruits were bought from a local vendor in Bahawalpur (city in Pakistan). Olives (*Olea europaea* L.) were procured from Allah Wala Farm in Bahawalpur. These raw materials were taken from their respective places to Cholistan University of Veterinary and Animal Sciences, Bahawalpur.

### 2.2. Chemicals and Reagents

DPPH (2,2‐diphenyl‐1‐picrylhydrazyl), quercetin, ascorbic acid, gallic acid, and Folin–Ciocalteu reagent were procured from Merck KGaA (Darmstadt, Germany). Chemicals such as sodium nitrite, trichloroacetic acid, potassium persulfate, sodium carbonate, aluminum chloride, ferric chloride, potassium ferricyanide, and potassium phosphate of analytical grade were procured from Sigma‐Aldrich (Castle Hill, New South Wales, Australia). Rennet and culture (CHR HANSEN, Denmark) were provided by United Food and Dairy Industry, Pirmahal.

### 2.3. Treatment Plan for the Manufacturing of Cheddar‐Type Cheese Supplemented With Olive Fruit Paste and Date Fruit Paste

Table 1 presents the treatment plan for the manufacturing of Cheddar cheese supplemented with olive fruit paste and date fruit paste. Cheddar cheese was prepared with varying concentrations of olive fruit paste (0%–6%), and 6% date fruit paste was also added in all treatments except T_0−_ (without containing olive fruit paste and date fruit paste). T_0+_ denotes Cheddar cheese containing 6% date fruit paste and 0% olive fruit paste. Treatments T_1_, T_2_, T_3_, and T_4_ refer to Cheddar cheese supplemented with 1.5%, 3%, 4.5%, and 6% olive fruit paste, respectively.

**Table 1:** Experimental treatment design of Cheddar‐type cheese supplemented with olive fruit paste.

Treatments	Cheese quantity	Olive fruit paste quantity	Date fruit paste quantity
T_0−_	1 kg	0%	0%
T_0+_	1 kg	0%	6%
T_1_	1 kg	1.5%	6%
T_2_	1 kg	3%	6%
T_3_	1 kg	4.5%	6%
T_4_	1 kg	6%	6%

*Note:* T_0−_ = control Cheddar cheese (without olive fruit paste and date fruit paste); T_0+_ = Cheddar cheese supplemented with 0% olive fruit paste and 6% date fruit paste; T_1_ = Cheddar cheese supplemented with 1.5% olive fruit paste; T_2_ = Cheddar cheese supplemented with 3% olive fruit paste; T_3_ = Cheddar cheese supplemented with 4.5% olive fruit paste; T_4_ = Cheddar cheese supplemented with 6% olive fruit paste.

### 2.4. Manufacturing of Cheddar Cheese

**Figure 1:**
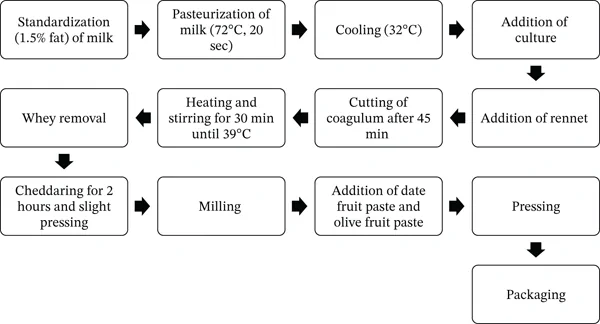
Processing steps for the manufacturing of Cheddar cheese supplemented with olive fruit paste and date fruit paste.

Cheddar cheese was prepared according to the method described in the study of Riebel et al. [7] with slight modifications. The processing steps for the manufacturing of Cheddar cheese supplemented with olive fruit paste and date fruit paste are presented in Figure 1. Following standardization of milk (1.5% fat), the milk was pasteurized at 72°C for 20 s and then cooled to 32°C through continuous stirring. The DVS culture (mesophilic) was added to the milk and kept for 20 min. Calcium chloride (0.05% w/v) was also added to speed up the coagulation process. After this, rennet was mixed with the milk and left until the milk was converted into coagulum. The coagulum was cut into small pieces, and the temperature of the coagulum was gradually elevated to 39°C with stirring. The coagulum was cooked/scalded for 30 min. The whey was removed by pouring it through muslin cloth. The cheddaring process was performed for 2 h, and the cheese matrix was slightly pressed so as to remove most of the whey. The milling process was carried out, and then date fruit pastes and olive fruit pastes were added to the cheese matrix according to our treatments (0%, 1.5%, 3%, 4.5%, and 6%). The cheese‐making process was carried out thrice so as to make three replicates of each treatment. Finally, cheese samples of all the treatments were pressed and packed for storage at 4°C.

### 2.5. Physicochemical Characteristics of Cheddar Cheese

The pH and titratable acidity of Cheddar cheese were measured as described by the standard procedure of AOAC [47]. Moisture, protein, fat, and fiber content of Cheddar cheese were also evaluated through the standard method described by AOAC [47].

### 2.6. Antioxidant Potential of Cheddar Cheese

#### 2.6.1. Preparation of Ethanol‐Soluble Extract (ESE) of Cheddar Cheese

Cheddar cheese sample (1.0 g) was extracted with 10 mL of ethanol (80%) with continuous stirring at ambient temperature. The samples were centrifuged (Hermle Labortechnik GmbH, Siemensstr‐25 D‐78564 Wehingen, Germany) at 10,000 rpm for 5 min at 4°C after keeping the samples for 24 h. Finally, samples were filtered using Whatman No. 1 filter paper. The ethanolic extract of Cheddar cheese was utilized for the assessment of total flavonoid content (TFC), ferric reducing antioxidant power (FRAP), DPPH, and total phenolic (TP) content [48].

#### 2.6.2. DPPH Radical Scavenging Activity

DPPH radical scavenging activity of Cheddar cheese was conducted following the previously reported method of Cho et al. [49]. Briefly, 0.5 mL of ESE of Cheddar cheese was mixed with 1 mL of distilled water, and then 1.5 mL of DPPH solution (0.1 mM) was added. The mixture was incubated for 30 min in the dark, and then absorbance was recorded at 517 nm using a UV‐Vis spectrophotometer (T80, PG Instrument). The results were expressed as milligrams of ascorbic acid equivalents per 100 g.

#### 2.6.3. FRAP

The FRAP of ESE of Cheddar cheese was determined as reported by Qureshi et al. [50] with slight modifications. Firstly, 0.5 mL of sample aliquot was mixed with 0.5 mL of potassium phosphate buffer (0.2 M, pH 6.6), and then 0.5 mL of potassium ferricyanide (0.5%) was added. After this, 0.5 mL of trichloroacetic acid (10%) was added to the mixture. The mixture was centrifuged (5000 rpm for 5 min at 4°C) to obtain a clear supernatant. Then, 0.2 mL of ferric chloride (0.1%) was added to the supernatant, and the absorbance of the mixture was determined using a UV‐Vis spectrophotometer (T80, PG Instrument) at 700 nm. The FRAP was measured as milligrams of ascorbic acid equivalents per 100 g of Cheddar cheese.

#### 2.6.4. TFCs

TFCs of ESE of Cheddar cheese were determined according to El‐Nawasany et al. [51]. An aliquot (0.5 mL) of ESE of Cheddar cheese was mixed with 0.1 mL of sodium nitrite (5%) solution and 0.2 mL of aluminum chloride (10%) solution. After this, 1 mL of NaOH (1 M) was added, followed by the addition of 1.5 mL of distilled water. The mixture was incubated for 30 min, and then absorbance was measured at 510 nm using a UV‐Vis spectrophotometer (T80, PG Instrument). Quercetin was utilized as a standard, and the results were expressed as milligrams of quercetin equivalent per 100 g.

#### 2.6.5. TP Content

The TP contents of ESE of Cheddar cheese were measured using a spectrophotometric method as reported by da Silva et al. [52]. 0.5 mL of ESE was mixed with 1 mL of Folin–Ciocalteu reagent (10%) and 1 mL of sodium carbonate solution (10%). Finally, the absorbance of the mixture was recorded at 760 nm using a UV‐Vis spectrophotometer (T80, PG Instrument) after 30 min incubation at room temperature. The TP of Cheddar cheese was quantified as milligrams of gallic acid equivalents per 100 g.

#### 2.6.6. Microbiological Assessment of Cheddar Cheese

Cheddar cheese was microbiologically assessed for total plate counts (TPCs, log colony‐forming units per gram of Cheddar cheese) and yeast and mold (Y&M, log colony‐forming units per gram) counts throughout the storage period described by Broadbent et al. [53]. Ten grams of Cheddar cheese was homogenized with 90 mL of saline water. One milliliter of different dilutions of the above suspension (up to 10^−5^) was then plated on plate count agar media (for TPC) and potato dextrose agar (for Y&M). After this, the plates were incubated for 24 h at 37°C in an incubator. After incubation, colonies on agar plates were counted and then multiplied by the dilution factor. The results were expressed as colony‐forming units per gram of Cheddar cheese.

#### 2.6.7. Sensory Analysis of Cheddar Cheese

The prepared Cheddar cheeses were assessed for sensory characteristics such as appearance, flavor, texture, and overall acceptability using a 9‐point hedonic scale (9 = *extremely like*, 1 = *extremely dislike*) [54]. The approval of the evaluation was done by the Institutional Ethical Review Committee (Certificate No. 525) of Cholistan University of Veterinary and Animal Sciences, Bahawalpur (No. ORIC‐525). The sensory analysis of Cheddar cheese was assessed by 20 members, including students and faculty members of the Department of Food Sciences, Cholistan University of Veterinary and Animal Sciences, Bahawalpur, who were familiar with the scaling procedures for cheese samples. Cheddar cheese samples were coded with random three‐digit numbers and served at room temperature. Disposable plates, cups, plastic forks, toothpicks, drinking water, plain crackers (for palate cleansing), sensory evaluation forms, and pens were available for sensory evaluation. Informed written consent was obtained from all participants during the sensory evaluation of the product.

### 2.7. Statistical Analysis

Statistical analysis was performed through Minitab statistical software Version 22.2.0 (Minitab Inc., State College, Pennsylvania, United States). Two‐way ANOVA was performed, and a pairwise comparison test was applied using Tukey′s test at a significance level of *p* < 0.05.

## 3. Results and Discussion

### 3.1. Physicochemical Characteristics of Cheddar Cheese

Table 2 presents the results of physicochemical characteristics of Cheddar cheese supplemented with olive fruit paste. The pH and titratable acidity of Cheddar cheese showed significant variations (*p* < 0.05) among the treatments during the storage period (2 months). The highest value (5.52) of pH was found in treatment T_0+_, whereas the lowest (5.18) was observed in T_4_ at Day 0. Among the treatments, T_0−_ exhibited the highest pH (5.49) after 2 months of storage, whereas T_4_ showed the lowest value (4.95). The increase in acidity levels during subsequent treatments may be due to a higher concentration of acids in olive fruit paste. The acidity (%) of fresh Cheddar cheese samples was in the range 0.39%–0.82%, but after completion of 2 months of storage, the acidity reached up to the range 0.33%–0.89%. Generally, pH decreased, and titratable acidity increased in all the treatments throughout the storage interval (0–2 months) except treatment T_0−_. Our results regarding pH and acidity were consistent with the findings of Khan et al. [55] and Abbas et al. [56], who also found an increasing trend of acidity (%) and a decreasing trend of pH during the storage period. The decrease in pH of Cheddar cheese during storage was attributable to the transformation of lactose into lactic acid. Khaliq et al. [57] reported that the pH of cream cottage cheese declined during storage. The acidity of the Cheddar cheese increased with the increasing concentration of olive fruit paste because of the presence of phenolic compounds and organic acids that increase the activity of LAB and decrease the buffering capacity.

**Table 2:** Physicochemical characteristics of Cheddar cheese supplemented with olive fruit paste.

Treatments	Months	pH	Acidity (%)	Moisture (%)	Protein (%)	Fat (%)	Crude fiber (%)	Ash (%)
T_0−_	0	5.31 ± 0.04c–e	0.39 ± 0.01kl	42.79 ± 1.12a	34.09 ± 0.17a	16.52 ± 0.09a	0.00 ± 0.00j	1.50 ± 0.09b
	1	5.42 ± 0.12a–c	0.37 ± 0.03kl	42.54 ± 1.28a	34.18 ± 0.77a	16.57 ± 0.39a	0.00 ± 0.00j	1.52 ± 0.08b
	2	5.49 ± 0.04a	0.33 ± 0.02l	42.40 ± 1.12a	34.29 ± 1.04a	16.59 ± 0.51a	0.00 ± 0.00j	1.56 ± 0.11b
T_0+_	0	5.52 ± 0.02a	0.42 ± 0.07jk	44.03 ± 1.51a	34.33 ± 0.50a	16.60 ± 0.45a	0.05 ± 0.00l	1.54 ± 0.13b
	1	5.47 ± 0.03ab	0.48 ± 0.01ij	43.51 ± 2.07a	34.35 ± 0.67a	16.66 ± 0.51a	0.06 ± 0.00l	1.54 ± 0.18b
	2	5.33 ± 0.05b–d	0.58 ± 0.04gh	43.14 ± 2.04a	34.39 ± 1.30a	16.48 ± 0.51a	0.06 ± 0.00l	1.61 ± 0.11b
T_1_	0	5.41 ± 0.04a–c	0.56 ± 0.09hi	44.50 ± 1.32a	34.42 ± 1.10a	16.65 ± 0.91a	0.170 ± 0.003h	1.59 ± 0.15b
	1	5.33 ± 0.06bc	0.58 ± 0.08gh	43.82 ± 1.91a	34.49 ± 0.79a	16.74 ± 0.72a	0.176 ± 0.005h	1.64 ± 0.10ab
	2	5.27 ± 0.045c–f	0.71 ± 0.07ef	43.22 ± 1.36a	34.59 ± 0.75a	16.74 ± 0.72a	0.190 ± 0.006h	1.68 ± 0.14ab
T_2_	0	5.33 ± 0.034bc	0.66 ± 0.01fg	45.23 ± 1.41a	34.55 ± 0.90a	17.09 ± 0.54a	0.26 ± 0.001g	1.63 ± 0.07ab
	1	5.17 ± 0.042e–h	0.78 ± 0.07c–e	45.10 ± 1.67a	34.70 ± 1.50a	17.23 ± 0.87a	0.29 ± 0.004f	1.70 ± 0.15ab
	2	5.11 ± 0.06g–i	0.80 ± 0.06b–d	44.83 ± 1.84a	34.82 ± 0.91a	17.23 ± 0.87a	0.31 ± 0.002f	1.79 ± 0.15ab
T_3_	0	5.25 ± 0.03d–g	0.72 ± 0.02d–f	45.83 ± 2.11a	34.61 ± 0.71a	17.49 ± 0.83a	0.37 ± 0.007e	1.69 ± 0.16ab
	1	5.12 ± 0.04f–i	0.79 ± 0.01b–e	45.34 ± 1.16a	34.81 ± 0.89a	17.71 ± 0.77a	0.41 ± 0.009d	1.78 ± 0.14ab
	2	4.97 ± 0.01ij	0.84 ± 0.02a–c	44.90 ± 1.53a	34.98 ± 1.21a	17.71 ± 0.77a	0.44 ± 0.006c	1.84 ± 0.08ab
T_4_	0	5.18 ± 0.04d–h	0.82 ± 0.008a–c	45.98 ± 1.79a	34.74 ± 0.59a	18.02 ± 0.75a	0.46 ± 0.003c	1.74 ± 0.08ab
	1	5.07 ± 0.07h–j	0.87 ± 0.03ab	45.56 ± 1.34a	34.89 ± 0.69a	18.13 ± 0.49a	0.49 ± 0.008b	1.82 ± 0.07ab
	2	4.95 ± 0.03j	0.89 ± 0.03a	44.92 ± 1.49a	35.03 ± 0.86a	18.13 ± 0.49a	0.52 ± 0.003a	1.99 ± 0.09a

*Note:* Data are presented as means ± standard deviation (*n* = 3). Means in the same column having different letters show significant (*p* < 0.05) variations between treatments and storage.

It is evident from the results that moisture contents were increased significantly (*p* < 0.05) by the addition of olive fruit paste in Cheddar cheese. The minimum moisture contents were recorded for T_0−_ (42.79%), whereas the maximum moisture contents were recorded for T_4_ (45.98%). During the storage period of Cheddar cheese, the moisture contents decreased among all the treatments. After 2 months, the highest (44.92%) moisture content was observed in treatment T_4_, and the lowest (42.40%) moisture content was found in T_0−_.

The analysis of variance revealed that crude fiber and crude fat content of Cheddar cheeses showed significant (*p* < 0.05) variations among the treatments. The fat contents were the maximum in T_4_ (18.02%), and the minimum fat contents were in T_0−_ (16.52%) on Day 0. The increasing trend of fat contents in subsequent treatments may be attributed to the higher concentration of fat/lipid content in olive fruit paste. Therefore, the addition of more and more olive fruit paste in Cheddar cheeses may be the reason for the increasing trend of fat content. The highest and lowest values for fiber contents of Cheddar cheese were found in T_4_ (0.46%) and T_0−_ (0.00%), respectively. The increasing trend of fiber content in subsequent treatments might be due to the presence of a higher concentration of fiber content in olive fruit paste.

There was a significant effect (*p* < 0.05) of treatments and storage period on the ash contents of Cheddar cheese. The maximum (1.74%) ash contents were observed in T_4_, whereas the minimum (1.50%) ash contents were found in T_0−_. The ash contents in Cheddar cheese increased with the increase in concentration of olive fruit paste. In addition, a slight decrease in moisture contents causes a slight increase in dry matter, which in turn may cause an increase in ash during storage. After 2 months, the lowest value (1.56%) of ash was shown by T_0−_, and the highest (1.99%) was found in T_4_. The protein contents of Cheddar cheese showed a slight change among all the treatments throughout the storage period, which might be due to the lower concentration of protein content in olive fruit paste. In addition, a slight decrease in moisture contents causes a slight increase in dry matter, which in turn may cause an increase in protein during storage.

The observed reduction in moisture contents of Cheddar cheese during storage was aligned with the results reported by Khaliq et al. [57] and Khan et al. [55]. The moisture contents of freshly prepared Cheddar cheeses in this research were higher than the moisture contents of Cheddar cheese reported by Chen et al. [58], Nadeem et al. [59], and Poudel et al. [9]. By the subsequent incorporation of olive fruit paste into the cheese matrix, moisture contents in Cheddar cheese were increased due to the presence of greater moisture contents in olive fruits. Such an increase in moisture contents in olive paste‐enriched cookies was also reported by Argyri et al. [60].

Our results concerning fat, protein, and ash contents of Cheddar cheese supplemented with olive fruit paste were consistent with the findings of Irfan et al. [61], who also observed changes in fat, protein, and ash content with the addition of olive oil emulsion in Cheddar cheese. In another study, the addition of extra virgin olive oil (EVOO) also gives a similar change in proximate composition of cream cottage cheese spread [57]. The fat content (%) of freshly prepared Cheddar cheese (T_0−_) in the current study was 16.52%, which was less than the fat content of Cheddar cheese (24.01%) reported by Nadeem et al. [59]. Lee et al. [62] also reported a higher fat content (20.25%) of Cheddar cheese (control) in comparison to our study. The main cause of these variations is the standardization of fat in milk at 1.5% before cheese making. There was an increasing order of ash, fat, protein, and fiber content of Cheddar cheese during storage as assessed in the current study. The increase in protein, ash, fat, and fiber contents during storage may be due to the increasing of dry matter (moisture content decreased), as reported by Nadeem et al. [59].

### 3.2. Microbial Counts of Cheddar Cheese

**Figure 2:**
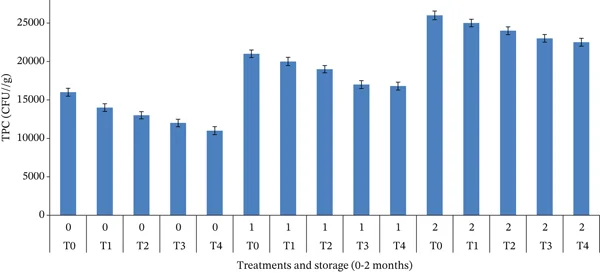
Total plate counts (TPCs, colony‐forming units per gram) of Cheddar cheese supplemented with olive fruit paste.

**Figure 3:**
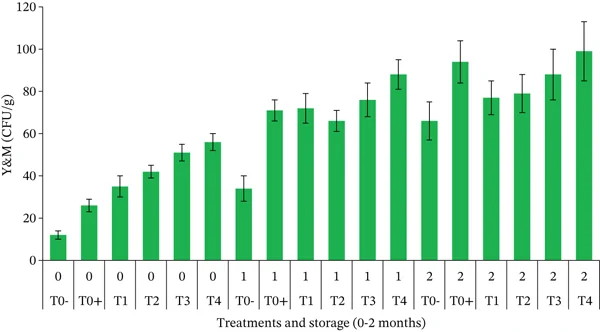
Yeast and mold (Y&M, colony‐forming units per gram) counts of Cheddar cheese supplemented with olive fruit paste.

Figure 2 shows the TPCs, whereas Figure 3 presents Y&M counts of Cheddar cheese supplemented with olive fruit paste. After 2 months of storage, the value of TPC of Cheddar cheese without containing date fruit and olive fruit paste (T_0−_) decreased from 2.2 × 10^4^ to 1.72 × 10^4^ CFU/g. Regarding Y&M counts, values of Cheddar cheese without containing date fruit and olive fruit paste (T_0−_) increased from 1.2 × 10^1^ to 6.6 × 10^1^ CFU/g. The TPC of control cheese (without olive fruit paste, T_0+_) was 1.6 × 10^4^ CFU/g which continued to increase significantly (*p* < 0.05), reaching 2.6 × 10^4^ CFU/g after 2 months of storage, whereas Cheddar cheese supplemented with olive fruit paste showed slightly lower values. The Y&M counts of control cheese (without olive fruit paste, T_0+_) were also significantly increased from 4.8 × 10^3^ to 9.36 × 10^3^ CFU/g. The lower TPC (1.1 × 10^4^ CFU/g) and Y&M counts (2.64 × 10^3^ CFU/g) were observed in T_4_ on Day 0 and after 2 months. TPC and Y&M counts reached up to 2.25 × 10^4^ and 8.1 × 10^3^ CFU/g, respectively. The TPC of all the prepared cheeses increased during storage. These results were consistent with the findings of Qureshi et al. [63]. Many studies reported the antimicrobial activity of olive fruit [25, 64, 65]. Hadjer et al. [66] reported a decrease in microbial counts in pasteurized white cheese with the addition of olive pomace extract. The slight decreasing trend of TPC in subsequent treatments of freshly prepared cheeses might be due to antimicrobial activities of olive fruit pastes.

### 3.3. Sensory Characteristics of Cheddar Cheese

**Figure 4:**
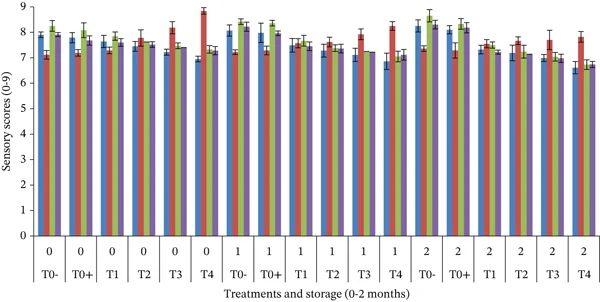
Sensory evaluation scores of Cheddar cheese supplemented with olive fruit paste; blue bars show appearance, red bars show flavor, green bars show texture, and purple bars show overall acceptability.

Figure 4 shows sensory evaluation scores of Cheddar cheese supplemented with olive fruit paste. The Cheddar cheeses prepared in the present study were assessed for texture, appearance, flavor, color, and overall acceptability during the storage period (0–2 months). It was observed that the appearance and texture scores of Cheddar cheese varied significantly (*p* < 0.05) due to the addition of more olive fruit paste. The texture and appearance scores of Cheddar cheese decreased with the increase in the quantity of olive fruit paste. The highest score of appearance (7.9) and texture (8.25) was observed in freshly prepared T_0−_ (Cheddar cheese having 0% olive fruit paste and 0% date fruit paste), whereas the lowest appearance (6.95) and texture (7.33) scores were obtained for freshly prepared T_4_ (Cheddar cheese having 6% olive fruit paste) on Day 0. The addition of olive fruit paste into Cheddar cheese changes the color (light greenish). All the freshly prepared Cheddar cheeses (T_0−_ to T_4_) were within acceptable limits regarding appearance and texture attributes. The texture and appearance scores of all the prepared Cheddar cheeses were significantly (*p* < 0.05) decreased during storage except control cheese samples (T_0−_ and T_0+_). The decreased texture score of Cheddar cheese with increasing quantities of olive fruit paste might be due to the presence of fibrous content in olives, resulting in a reduction of the smoothness of Cheddar cheese.

The results of the flavor of Cheddar cheese revealed that fresh Cheddar cheese supplemented with olive fruit paste was much liked by the panelists. The flavor of Cheddar cheese was enhanced with the increasing quantities of olive fruit paste. The highest flavor score (8.84) was shown by T_4_, whereas the lowest score (7.12) was found in T_0−_. The flavor and overall acceptability scores of all the prepared Cheddar cheeses supplemented with olive fruit paste were significantly (*p* < 0.05) decreased throughout the storage period (0–2 months). It was also observed that the overall acceptability of all Cheddar cheeses supplemented with olive fruit paste was not as acceptable as the control cheese samples (T_0−_ and T_0+_) after 2 months of storage.

This storage behavior of all prepared Cheddar cheeses supplemented with olive fruit paste (T_1_–T_4_) regarding appearance, flavor, texture, and overall acceptability was in accordance with the findings of Alqahtani et al. [67], who observed a decrease in appearance, texture, flavor, and overall acceptability of block‐type processed cheese during storage. The change in sensorial properties of Cheddar cheese during storage might be due to the many biological and metabolic processes (proteolysis, lipolysis, and glycolysis), resulting in the conversion of different compounds [68–71]. Irfan et al. [61] also observed a decrease in texture, appearance, and overall acceptability scores with the addition of olive oil emulsion.

### 3.4. Antioxidant Potential of Cheddar Cheese

**Figure 5:**
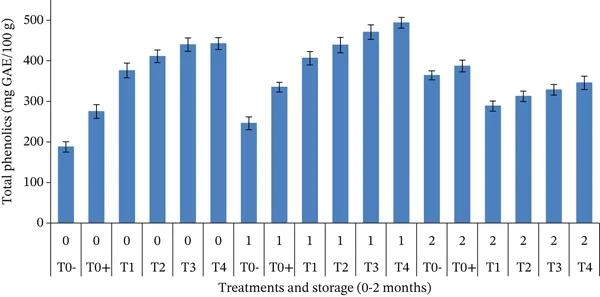
Total phenolics (milligrams of gallic acid equivalent per 100 g) of Cheddar cheese supplemented with olive fruit paste.

**Figure 6:**
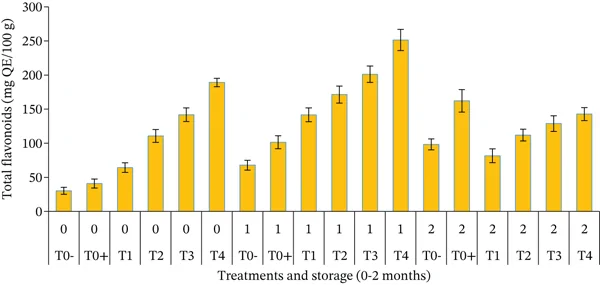
Total flavonoids (milligrams of quercetin equivalent per 100 g) of Cheddar cheese supplemented with olive fruit paste.

**Figure 7:**
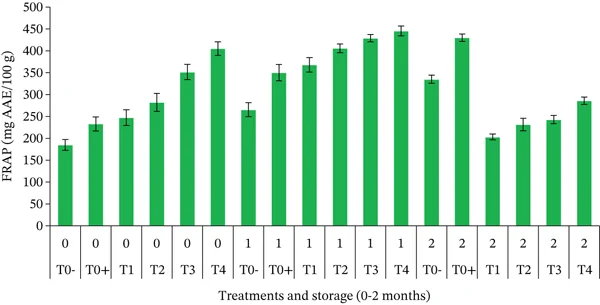
Ferric reducing antioxidant power (FRAP) (milligrams of ascorbic acid equivalent per 100 g) of Cheddar cheese supplemented with olive fruit paste.

**Figure 8:**
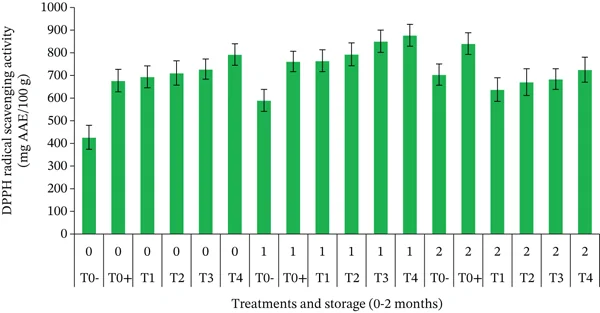
DPPH radical scavenging activity (milligrams of ascorbic acid equivalent per 100 g) of Cheddar cheese supplemented with olive fruit paste.

Figures 5, 6, 7, and 8 show the TPs, total flavonoids (TFs), FRAP, and DPPH activity of Cheddar cheese, respectively. The values of TP of freshly prepared Cheddar cheese (control) T_0−_ showed the minimum values (187.66 mg GAE/100 g), whereas the maximum values (442.47 mg GAE/100 g) were demonstrated by T_4_ (Cheddar cheese supplemented with 6% olive fruit paste) at Day 0. Therefore, the addition of more and more quantities of olive fruit paste caused an increase in TP in freshly prepared Cheddar cheese. The freshly prepared Cheddar cheeses incorporated with olive fruit paste showed higher TP values compared with control Cheddar cheese samples (T_0−_ and T_0+_). The TP values of all prepared Cheddar cheeses decreased gradually during storage. After 2 months, the highest values of TP were obtained in T_0+_ (387.13 mg GAE/100 g), and T_4_ showed a value of 345.67 mg GAE/100 g, which was lower than the control (T_0−_). The decrease in TP content of Cheddar cheese supplemented with olive fruit paste (T_1_–T_4_) after 2 months might be due to the degradation of phenolic compounds over the extended storage period. This suggests that freshly prepared Cheddar cheese supplemented with olive fruit paste had more phenolics until the 1st month of storage, and then phenolic contents were in a decreasing trend, whereas Cheddar cheese (control) was in an increasing trend over the storage period. The decrease in phenolic contents might be due to the degradation of glycoside forms of phenolic compounds to the aglycone forms by the action of enzymes [72].

There were significant (*p* < 0.05) effects of treatments on the TFC of Cheddar cheese during the storage period. The TFC of Cheddar cheese (T_1_–T_4_) decreased significantly after 2 months of storage, whereas TFC in control Cheddar cheese samples (T_0−_ and T_0+_) subsequently increased over the storage period. The highest TFC was observed in T_4_ (189.13 mg/100 g), and the lowest was found in T_0−_ (30.23 mg/100 g) on Day 0.

There were significant (*p* < 0.05) differences in DPPH and FRAP activity of Cheddar cheese among treatments and storage period. The DPPH value of fresh Cheddar cheese (control, T_0−_) showed the lowest value (426.87 mg/100 g AAE) as compared with Cheddar cheese (olive fruit paste supplemented), whereas the highest value (792.73 mg/100 g AAE) was obtained by fresh Cheddar cheese supplemented with 6% olive fruit paste (T_4_). The DPPH activity of all prepared Cheddar cheeses increased until the 1st month of storage, and then a change was observed after 2 months of storage. Upon the completion of 2 months, T_0+_ showed the maximum value (840.67 mg/100 g AAE) of DPPH compared with other Cheddar cheese supplemented with olive fruit paste (T_1_–T_4_). The DPPH activity of Cheddar cheese (control) showed an increasing trend throughout the storage period (0–2 months), whereas the Cheddar cheese (olive fruit paste supplemented) had a decreasing trend in DPPH after 2 months.

Significant differences (*p* < 0.05) were observed in FRAP activity among treatments during the storage period. The FRAP activity of all the Cheddar cheese treatments increased until the 1st month of the storage period, but afterwards, the FRAP activity of Cheddar cheeses (olive fruit paste supplemented) started to decrease. The minimum activity (185.02 mg/100 g AAE) of FRAP was observed in freshly prepared Cheddar cheese (control, T_0−_), and the highest (405.27 mg/100 g AAE) was found in T_4_ on Day 0. The FRAP values of Cheddar cheese increased with the corresponding increase in olive fruit paste concentration. After 2 months, the maximum (430.05 mg/100 g AAE) FRAP activity was observed in control Cheddar cheese (T_0+_) after 2 months as compared with other treatments (T_0−_, T_1_–T_4_).

The TPs and flavonoid content of Cheddar cheeses (T_0+_–T_4_) increased up to the 1st month of storage, and then a change was observed after 2 months of storage. After 2 months, cheese supplemented with olive fruit paste (T_1_–T_4_) showed a decreasing trend of TPs and TFs, whereas control cheese samples (T_0−_ and T_0+_) demonstrated an increasing concentration of TPs and TFs over the storage period (0–2 months). The results of our control cheese (T_0−_) corresponded with the findings of Nadeem et al. [59], who similarly noted that the phenolic and flavonoid content of Cheddar cheese increased during the ripening (storage) period.

This study also found an increasing concentration of TPs and TFs with the addition of olive fruit paste in Cheddar cheese. These findings were in line with the results of Irfan et al. [61], who observed an increase in the quantities of flavonoids and phenolics with the addition of olive oil emulsion in processed cheese. Khaliq et al. [57] also observed that TPs increased with the increase in the quantities of EVOO in cream cottage cheese. In another study, yoghurt enriched with olive pomace had higher antioxidant activity and TP content [73]. Carboni et al. [45] also observed an increase in TPs and antioxidant capacity of sheep milk yoghurt after incorporation of olive pomace powder. Based on the findings of previous studies, it was concluded that olive fruit by‐products (olive oil and olive pomace) enhanced the antioxidant activity and also increased TPs and TFs in dairy products.

The DPPH and FRAP activity of Cheddar cheeses increased at the start of the storage (ripening) period and then subsequently decreased over prolong storage period, as reported in the research conducted by Chen et al. [58]. The FRAP and DPPH activity of Cheddar cheese may be due to the presence of bioactive peptides derived from *β*‐casein, namely, *κ*‐casein, *α*_s1_‐casein, and *α*_s2_‐casein, in the cheese matrix which are typically released during the ripening period [74–78]. The reducing power and scavenging activity of Cheddar cheese were also decreased mainly due to the production of smaller peptides (less effective) during extensive proteolysis (degradation of protein), thereby resulting in a decrease in total antioxidant activity [79]. The DPPH and FRAP activity of all the prepared Cheddar cheeses increased up to the 1st month of storage, and then a change was observed after 2 months. Control cheese (without olive fruit paste) showed an increase in DPPH and FRAP activity throughout the storage period, whereas cheese supplemented with olive fruit paste showed a decline after the 1st month of storage. This suggests that Cheddar cheese supplemented with olive fruit paste was degraded over prolong storage period (2 months) which might be due to the degradation of phenolic compounds as a result of oxidation [80] and enzymatic activity [72], hence resulting in the decrease in the antioxidant potential of Cheddar cheese. When olive fruit begins to rot as a result of microbial activity, the degradation of oleuropein is one of the first processes to occur, resulting in an increase in its degradation products, but over time, these products also begin to decrease [81]. Burin et al. [80] also observed that phenolic compounds of olive fruits decreased after 16 days of storage period due to deterioration. This also indicates that the investigated Cheddar cheese supplemented with olive fruit paste showed a decreasing trend of antioxidant activity during storage. The antioxidant activity of freshly prepared cheeses supplemented with olive fruit pastes was increased due to the subsequent increase in the quantities of olive fruits. At the start, there was no deterioration of olive paste inside the cheese matrix. The decreased shelf stability or deterioration of Cheddar cheese might be due to undesirable microbial activities in the cheese matrix during storage owing to the presence of more moisture from olive and date fruit pastes.

## 4. Conclusion

Based on our findings, it was concluded that physicochemical attributes (pH, ash content, titratable acidity, crude fiber, fat content, and moisture content) of Cheddar cheese showed significant (*p* < 0.05) variations among the treatments during storage. The texture and appearance score of fresh Cheddar cheese decreased with the increase in quantities of olive fruit pastes, whereas the flavor score increased with the subsequent addition of olive fruit paste (T_1_–T_4_). The sensory scores of cheese incorporated with olive fruit paste (T_1_–T_4_) decreased during storage. It was also observed that freshly prepared Cheddar cheese supplemented with 6% olive fruit paste (T_4_) showed the highest phenolic contents, TFCs, DPPH activity, and FRAP activity. Cheddar cheese supplemented with olive fruit paste showed a decreasing trend of TPs, TFs, FRAP activity, and DPPH activity during storage, whereas control cheese samples (without olive fruit paste, T_0−_ and T_0+_) showed an increasing trend of antioxidant potential throughout the storage (0–2 months). The maximum TPCs and Y&M counts were found in T_0+_ (control cheese), whereas the minimum TPC and Y&M counts were observed in T_0−_ after 2 months of storage. It was concluded that freshly prepared Cheddar cheese supplemented with olive fruit paste would be nutritious and healthy due to the greater antioxidant activity compared with the control cheese samples (without olive fruit paste, T_0+_ and T_0−_).

The findings suggest that olive fruit paste serves as a natural functional ingredient for the development of highly nutritious and value‐added dairy products. The utilization of olive fruit paste in the dairy industry may support the production of innovative functional cheese with improved nutritional and sensory properties.

Future studies should emphasize the quantification of phenolic compounds after the addition of olive fruit powder in Cheddar cheese.

## Author Contributions

**Munahil Mahmood:** formal analysis, data curation, writing – original draft. **Tahir Mahmood Qureshi:** conceptualization, supervision, investigation, visualization, validation, software, methodology, writing – review and editing. **Mehrooz Ishfaq:** formal analysis, investigation, data curation, visualization, writing – original draft. **Rana Muhammad Bilal:** conceptualization, validation, writing – review and editing. **Muhammad Nadeem:** conceptualization, writing – review and editing. **Haile Welearegay:** validation, methodology, writing – review and editing. **Mestawet Taye:** validation, writing – review and editing.

## Funding

No funding was received for this manuscript.

## Conflicts of Interest

The authors declare no conflicts of interest.

## Data Availability

The data that support the findings of this study are contained in the manuscript.
